# The role of E-Cadherin expression in primary site of breast cancer

**DOI:** 10.1007/s00404-021-06198-1

**Published:** 2021-09-12

**Authors:** Nora Karsten, Thomas Kolben, Sven Mahner, Susanne Beyer, Sarah Meister, Christina Kuhn, Elisa Schmoeckel, Rachel Wuerstlein, Nadia Harbeck, Nina Ditsch, Udo Jeschke, Klaus Friese, Theresa Maria Kolben

**Affiliations:** 1 Department of Obstetrics and Gynaecology, Breast Center and CCCLMU, LMU University Hospital, Marchioninistr. 15, 81377 Munich, Germany; 2 Department of Pathology, LMU Munich, Marchioninistr. 27, 81377 Munich, Germany; 3 Department of Gynaecology and Obstetrics, University Hospital, Stenglinstr. 2, 86156 Augsburg, Germany; 4 Department of Oncology, Hospital Bad Trissl, Bad-Trissl-Straße 73, 83080 Oberaudorf, Germany

**Keywords:** E-cadherin, Breast cancer, Recurrence, Metastasis, Prognosis

## Abstract

**Purpose:**

The tumour’s ability to metastasize is the major cause for fatal outcomes in cancer diseases. In breast cancer, aberrant E-Cadherin expression has been linked to invasiveness and poor prognosis.

**Method:**

We assessed expression of E-Cadherin by immunohistochemistry in primary tumour tissue from 125 female breast cancer patients. Staining intensities were analysed using the immunoreactive score (IRS). We investigated E-Cadherin expression and its associations with clinicopathological parameters (age, tumour size, lymph node status, grade, hormone receptors, Her2 Status) as well as with recurrence and survival.

**Results:**

Increased, rather than aberrant E-Cadherin expression was found and was associated with poor outcome (*p* = 0.046). Our data show an association between elevated E-Cadherin in primary tumour tissue and an unfavourable negative prognosis in patients.

**Conclusion:**

This association was somehow unexpected as loss of E-Cadherin has long been regarded as a prerequisite for development of invasiveness and metastases. Our findings support the notion that E-Cadherin promotes, rather than suppresses, development of metastasis and invasiveness.

## Introduction

With a worldwide incidence of 1.67 million patients per year, breast cancer is the most common cancer and with about half a million deaths per year, the leading cause of mortality in women worldwide [1]. Even though the incidence of breast cancer remains very high, the mortality rate has declined over the past 20 years [2]. This is mainly due to improved treatment options, which have developed from conventional locoregional to systemic targeted anti-tumour therapies. Depending on tumour subtype, surgery, radiotherapy, and systemic treatments such as endocrine therapy, targeted treatment, and chemotherapy are key treatment options [3].

The ability of cancer cells to migrate from the primary tumour site and form metastases is one of the hallmarks of cancer and the leading cause of death in cancer patients [4]. One postulated explanation for this ability is the assumption that single tumour cells emigrate individually from the primary tumour site mediated by processes called epithelial-mesenchymal transition (EMT). Individual cell movement is one of the best-studied cell movement mechanism [5].

During the cellular process of EMT epithelial cells lose their epithelial features and acquire mesenchymal characteristics [6]. EMT has been associated with loss of the cell-to-cell adhesion molecule E-Cadherin and gain of the mesenchymal marker Vimentin [7]. It has recently been postulated that EMT might not simply be a binary process, as previously assumed, but a more complex process in which cells go through different developmental transition states, which are defined by various types of epithelial and mesenchymal markers [8].

E-Cadherin, a member of the Cadherin-superfamily, is a calcium-dependent transmembrane glycoprotein, first described in 1977 by Takeichi [9]. A type-1-cadherine, E-Cadherin has multiple roles in physiological as well as pathological processes of cell migration and invasion, such as embryonic development [10], tissue morphogenesis [11] and cell–cell adhesion between neighbouring epithelial cells [12]. The role of E-Cadherin in cancer progression is established and well documented [13–16], represented by repression of E-Cadherin expression at the primary tumour site [17, 18]. E-Cadherin has been classified as a tumour suppressor and diminished E-Cadherin expression in epithelial cancer cells has been related to the process of EMT in multiple carcinomas, including breast cancer [19, 20] and to the acquisition of chemoresistance [21, 22].

The purpose of this study was to evaluate the relevance of E-Cadherin expression in tumour tissue at different stages, in the punch biopsies before any treatment and at the time of surgery, in patients with breast cancer. To our knowledge, little data exists concerning E-Cadherin as a marker involved in cancer development for prediction of outcome in patients. Therefore, we evaluated E-Cadherin expression and its associations with clinicopathological parameters as well as patient outcome.

## Materials and methods

### Patients

**Fig. 1:**
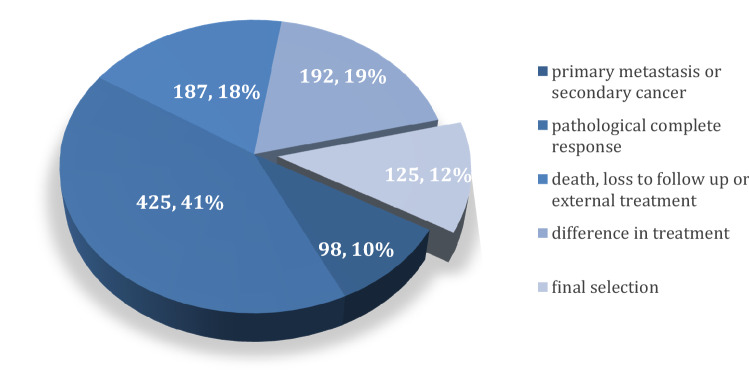
Flowchart demonstrating data from 1027 patients with primary breast cancer that had been registered at the breast center of the LMU University Hospital between 2005 and 2015 and had been considered for inclusion in this study. From these 1027 patients, we had to exclude 98 patients because of either primary metastasis or secondary cancer diseases. From the remaining 929 breast cancer patients, 425 patients with pathological complete response (pCR) had been excluded. Further on, because of unfinished treatment due to death, loss to follow-up or continued external treatment we had to exclude 187 more patients, leaving 317 for further analysis

In this study, we included patients with primary breast cancer diagnosis, i.e. non-metastatic disease, who were diagnosed and treated between 2005 and 2015 at the Breast Center, Department of Gynaecology and Obstetrics, Ludwig-Maximilians-University of Munich, Germany. Patients with primary metastatic disease were excluded as well as patients with pathological complete response (pCR) after neoadjuvant chemotherapy since we aimed to analyse and compare tumour tissue in the punch biopsy and at time of surgery. Data from 1027 patients with primary breast cancer that had been registered at the breast center of the LMU University Hospital between 2005 and 2015 had been considered for inclusion in this study. Of these, 125 patients with sufficient tumour tissue still available for analysis who met these criteria were identified. For the inclusion and exclusion process (see Fig. 1). Of the 125 selected breast cancer patients 95 patients had either a punch biopsy tissue (*N* = 62) at time of initial diagnosis or a surgical specimen (*N* = 78) or both (*N* = 45). Ethical compliance of the study was approved by the institutional review board of Munich, Germany (number: 17-819).

Demographic data including age at diagnosis, tumour grade, TNM Status, Hormone receptor status (progesterone and estrogen receptor status) and Her2 Status were retrieved from the Munich Cancer Registry or the Institute of Pathology, Ludwig-Maximilians-University of Munich. Patients were contacted via phone to collect information about present progression- and survival information. Recurrence status could be assessed in 104 out of 125 patients. Of these 104 patients, 9 (8.7%) had experienced a recurrence event, whereas 95 (91.3%) were still recurrence-free at time of analysis. The median follow-up period in years was 7.86 (see Table 1).

**Table 1:** Clinical and pathological parameters of patients included in this study

Age	Mean	Range
	54.4	28–81

	Frequency	Valid percent
Histology		
NST	110	88.7
Non-NST	14	11.3
Missing	1	
pTNM		
T1a	4	3.7
T1b	6	5.6
T1c	25	23.1
T2	51	47.2
T3	20	18.5
T4	2	1.9
Missing	17	
pN0	59	49.2
pN1-pN3	61	50.8
Missing	5	
Hormone receptor		
Positive	82	65.6
Negative	43	34.4
Missing	0	
Her2 Status		
Positive	19	15.7
Negative	102	84.3
Missing	4	
Recurrence		
No recurrence	95	91.3
Recurrence	9	8.7
Missing	21	
Type of treatment		
Primary surgery	57	45.6
Neoadjuvant chemotherapy	68	54.4
Adjuvant endocrine therapy	24	19.2
(intra-/postsurgical radiation)	93	74.4
Adjuvant chemotherapy	108	86.4
Tumour grade		
G1	12	9.9
G2	61	50.4
G3	48	39.7
Missing	4	

### Immunohistochemistry

**Fig. 2:**
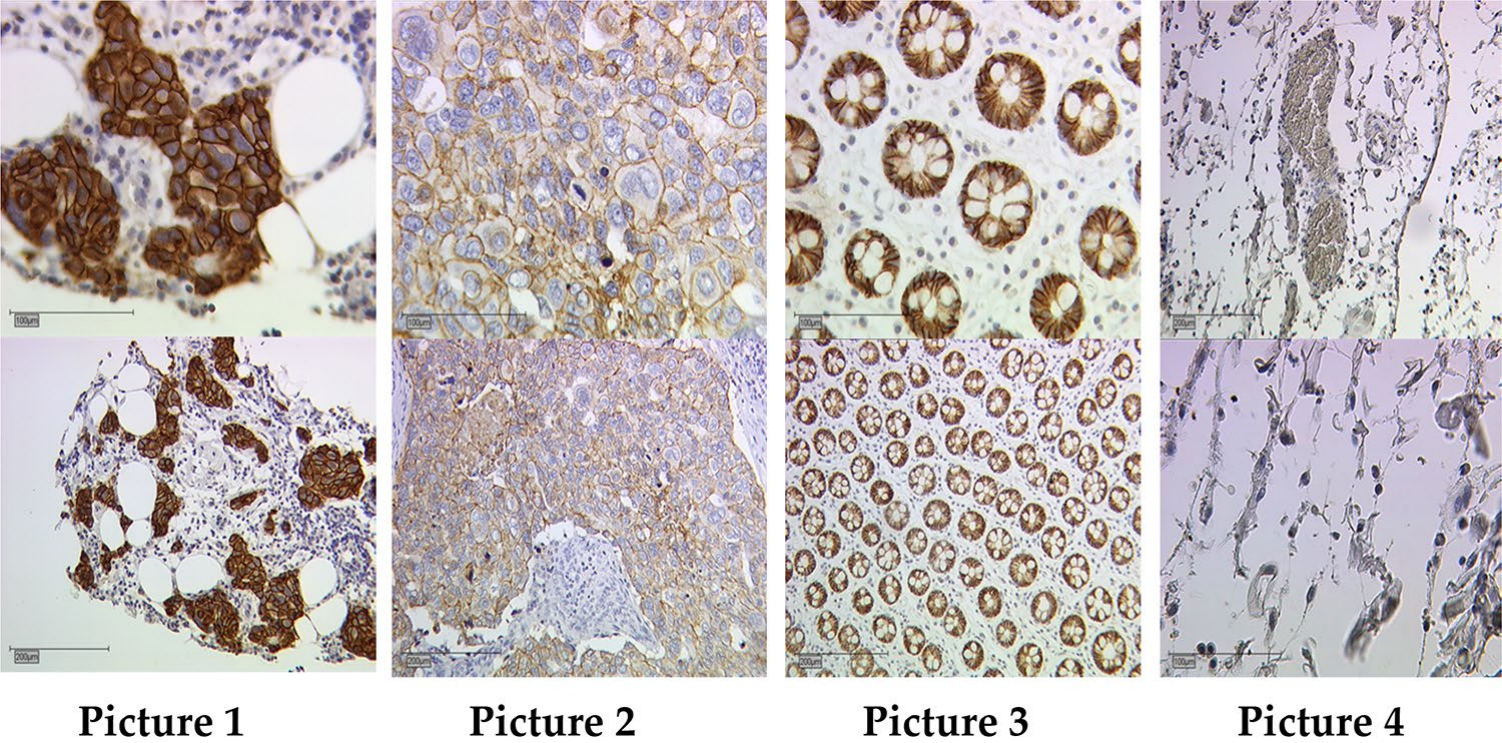
E-Cadherin staining in breast cancer. E-Cadherin IRS was scored with 12 in picture 1 (high intensity, ≥ 80% stained cells), 25 × magnified (top) and 10 × magnified (bottom). Picture 2 demonstrating an E-Cadherin IRS of 4 (low intensity, ≥ 80% stained cells), 25 × magnified (top), 10 × magnified (bottom). Picture 3 and 4 showing positive and negative controls (colon tissue), 25 × magnified (top), 10 × magnified (bottom)

Punch biopsy as well as surgical tissue was used for analysis. Tissue specimens were formalin-fixed and paraffin embedded before sectioned into 10 µm slices. Expression of E-Cadherin was assessed by immunohistochemistry. E-Cadherin staining was performed as described previously [23]. Primary anti-E-Cadherin antibody (monoclonal mouse IgG1, Abcam, Cambridge, UK), was used for tissue slide staining. Detection was performed via polymer-method (ZytoChem Plus HRP Polymer System (Mouse/Rabbit); Zytomed Systems Berlin, Germany; Nr. POLHRP-100) and chromogen diaminobenzidine (Dako, Hamburg, Germany) (See Fig. 2 for example stainings).

Colon tissue was stained simultaneously and used as a positive control. Expression of E-Cadherin was then assessed by the semi-quantitative immunoreactivity score (IRS) using a Leitz (Wetzlar, Germany) microscope. The IRS is the product of the intensity of the staining (0 = no staining, 1 = weak staining, 2 = moderate staining, 3 = strong staining) multiplied by the percentage of positive cells (0 = no staining, 1 =  < 10% positive cells, 2 = 11–50% positive cells, 3 = 51–80%, 4 =  > 81% positive cells). This multiplication has a minimum of 0 and a maximum of 12. Samples were then categorized as E-Cadherin positive and E-Cadherin negative, according to their IRS: Samples with an IRS score of 1 or higher were counted as E-Cadherin positive, whereas samples with scores of 0 were categorized as E-Cadherin negative. Mean ranks of the IRS scores were calculated using SPSS. For further detail, see section “statistical analysis”. We then compared E-Cadherin expression in samples from punch biopsies and surgical tissue samples. E-Cadherin expression was then correlated with clinicopathological parameters, progression-free and overall survival.

Estrogen and progesterone receptor status was categorized as positive when the percentage of positive tumour cells stained for estrogen and progesterone was at least 1%. The Her2 Status was defined as amplified with a FISH to ratio of higher than 2.2 [24] or overexpressed with an immunohistochemistry score of 3 + (membrane staining of 30% of tumour cells) [25].

### Statistical analyses

The statistical analysis was performed using the IBM SPSS software version 25. *P* values ≤ 0.05 were considered statistically significant. Chi-squared tests were used to determine independence between nominal data. For non-parametric data Kruskal–Wallis tests and Mann–Whitney *U* tests were used to assess relationships among clinicopathological parameters, antibody expression and type of treatment. Progression-free survival and overall survival curves were plotted using the Kaplan–Meier method. Since the data were not normally distributed we used the Kruskal–Wallis test to test for differences between several independent groups. This test is based on ranked data. IRS scores were ordered from lowest to highest; the lowest score was assigned a rank of 1, the next highest score was assigned a rank of 2 and so on. Ranks for each group are then ranked and the mean rank calculated via SPSS. The sum of ranks has been calculated for each group, square the sum of ranks and divide this value by the sample size for that group. Test statistics were compared between the two groups.

## Results

### Patients’ characteristics

Our collective consisted of 125 primary breast cancer patients. Patients have been collected retrospectively. All therapies have been chosen clinically and this retrospective study did not interfere with treatment choice. Mean patient age was 54.4 years (28–81 years). 88.7% of all patients had a carcinoma of no special type (NST). HER2 showed to be amplified in 15.7% of patients. 49.2% of patients had no lymph node metastasis (N0), in 50.8% one or more lymph nodes were involved (N1 or higher). Approximately half of the patients (50.4%) had a G2 tumour. Type of treatment was almost equally distributed between the patients: Out of the 125 selected patients, 57 (45.6%) had received primary surgery (see Table 1).

### No difference of E-Cadherin expression between punch biopsy and surgical specimen

We compared E-Cadherin expression in tumour tissue of punch biopsies and surgical samples. We initially compared the mean ranks of E-Cadherin IRS of all core biopsies and surgical specimen. No significant differences in E-Cadherin expression were found. This remained the case, when we compared the paired samples (45 pairs of tumour tissue from core biopsies and surgical specimen), no significant differences were found (see Fig. 2).

### Associations between nodal status (pN) and expression of E-Cadherin

Information about the nodal status was obtained in 59 out of 62 patients with available core biopsies. We used the information of nodal status at the time of primary diagnosis, prior to any treatment. Of these, 34 patients showed no nodal involvement (N0), whereas 25 patients had a positive nodal status (N1 or higher). Tumour tissue from patients with a positive nodal status showed a significantly (*p* = 0.034) higher E-cadherin IRS (median rank: 35.42) than tissue from patients with a negative nodal status (median rank: 26.01). When looked at the nodal status as the dependent variable, we found that amongst N0 patients (*n* = 34), tumour tissue of 3 (8.8%) patients showed a negative E-Cadherin expression (IRS < 1), whereas tumour tissue of 31 (91.2%) patients showed a positive E-Cadherin expression with IRS higher than one. As for patients (with N1 or higher status (*n* = 25), only tumour tissue of one (4%) patient was found to have a negative E-Cadherin expression (IRS < 2), as compared to 24 (96%) patients with a positive E-Cadherin expression in their tumour tissue.

### Association between hormone receptor status and E-Cadherin expression

Hormone receptor status could be obtained in a total of 122 patients. Of these 122 tumours, 89 were estrogen receptor positive and 33 estrogen receptor negative, whereas 76 were progesterone receptor positive and 46 were progesterone receptor negative (see Table 1). A highly significant (*p* = 0.006) association of high E-Cadherin expression (median rank: 40.18) and negative estrogen receptor status was observed. In addition, high E-Cadherin expression (median rank: 36.77) also correlated significantly (*p* = 0.021) with negative progesterone receptor status.

### High E-Cadherin expression in punch biopsies is associated with more frequent recurrence events

Patients with tumour tissue with E-Cadherin IR scores 1 or higher in the punch biopsy had more recurrence events. This correlation was found to be statistically significant (*p* = 0.046). The strength of this association was even stronger when selectively analysing punch biopsy tissue with high E-Cadherin IRS (IRS > 7).

### Positive Her2 Status combined with positive E-Cadherin IRS is associated with shorter time to recurrence

Information about E-cadherin IRS of the punch biopsy tissues samples, Her2 Status and follow-up data concerning recurrence status and recurrence-free survival time was available in a total of 93 patients. Of these 93 patients, 86 remained recurrence-free during our follow-up time, whereas six patients suffered from a recurrence event. Our data showed a significant difference (*p* = 0.01) between patients with Her2 positive status and IRS higher than one compared to patients who had a negative Her2 Status and negative E-cadherin IRS when looked at recurrence-free survival time. 50% of all patients, with tissue that was Her2 status positive and E-Cadherin IRS > 1, suffered from a recurrence event within the first year after treatment with neoadjuvant chemotherapy. In comparison, the median time to recurrence for patients with negative Her2 Status and negative E-Cadherin immunoreactive scores was 7 years. Treatment did not differ between both groups (see Table 2).

**Table 2:** Comparison of median time to recurrence dependent on Her2 Status and E-Cadherin expression

	Her2 Status negative and E-Cadherin IRS negative	Her2 Status positive and E-Cadherin IRS positive
Median time to recurrence (in years)	1	7,3

### Influence of age on E-Cadherin expression

When we selectively analysed the 45 paired samples of punch biopsies and surgical samples, we found a correlation between age at diagnosis and E-Cadherin expression. Of the selected 45 paired samples, 33 patients were 50 years or older, whereas 12 patients were younger than 50 years. We could observe the trend that tumour tissue of patients older than 50 years had higher E-Cadherin expression in the surgical specimen. This observation was independent of tumour biology or treatment received. Tumour tissue of patients younger than 50 years showed a higher E-Cadherin expression in the punch biopsies.

## Discussion

In this study, we aimed to analyse E-Cadherin expression in tumour tissues of patients with breast cancer at different time points and to assess whether this E-Cadherin expression showed a correlation to currently established prognostic variables. Whereas we could not observe a statistically significant difference in E-Cadherin expression between punch biopsies and surgical specimens, our data demonstrated a correlation between E-Cadherin expression and hormone receptor status as well as nodal status and clinical outcome.

It has been shown that breast cancer patients with tumours that express hormone receptors have a reduced mortality [26]. About 65% of breast carcinomas express estrogen receptors, and these cases are usually associated with a better prognosis [27]. A relationship between the expression of E-Cadherin in tumour tissue and hormone receptor expression has been noted in several studies previously [28–31]. However, results of these studies vary. Some studies have demonstrated that lower E-Cadherin expression is associated with estrogen receptor negative breast carcinomas, whereas other studies have failed to confirm these findings. Our data demonstrated a strong correlation between a negative hormone receptor status and elevated E-Cadherin expression.

Similarly, reduced E-Cadherin expression has been shown to correlate with positive nodal status [32–34]. Our results demonstrated a strong relationship between elevated expression of E-Cadherin and development of nodal metastases. In addition, we observed that patients with tumours that show a higher E-Cadherin expression have a shorter time to recurrence when compared to patients with tumours with negative E-Cadherin expression. Although several studies have shown a correlation between E-Cadherin and negative prognostic factors such as nodal status and hormone receptor status, our results showing higher expression levels in these tumour tissues were somehow unexpected, as typically reduced E-Cadherin expression has been linked to invasiveness and poor prognosis [19, 20, 29–31, 35–41]. Nevertheless, these findings are also in line with results of a recent study and the notion that E-Cadherin promotes, rather than suppresses, development of metastasis. E-Cadherin expression levels were not only reported to be elevated, but also shown to be significantly associated with poor clinical outcome. Instead of suppressing metastasis, expression of E-Cadherin was found to function as a promotor of metastasis [8]. Other studies have also demonstrated a more complex role for E-Cadherin, as it has been shown that expression of E-Cadherin can be retained or even increased [40–47].

Through invasion cancer cells migrate and metastasize. Invasion can be conceptualized as a single cell process or a collective invasion of multiple coherent cancer cells. In collective invasion, cells invade distant organs cohesively as a multicellular unit. Characteristically, in collective invasion, tumour cells maintain their cell–cell adhesion molecules, like E-Cadherin. Growing evidence suggests that collective invasion plays a major role in tumour progression [8, 48–54]. Our results support the hypothesis that E-Cadherin seems to be involved in collective cells behaviour that lead to invasion and metastasis.

In summary, our results contribute to the growing body of evidence that indicates both pro- and anti-tumorigenic properties of E-Cadherin. The different potential roles for E-Cadherin in the pathogenesis of tumour progress remain of ongoing interest. Based on our data, and in line with recent previous studies, we conclude, that continued expression of cell adhesion molecules do not necessarily contribute to the suppression of tumour progression and metastases. Nevertheless, due to the limited sample size this observation warrants further validation in a larger patient cohort.
